# Bioactive ceramic-processed water modulates the gut microbiota and hepatic AMPK activation in *SMP30* knockout mice

**DOI:** 10.1017/gmb.2026.10018

**Published:** 2026-01-22

**Authors:** Dong-Hun Kim, Soo-Nyun Choi, Kyongman An, Ji-Hoon Kwak, Kyung-Seok Ko, Kyu-Shik Jeong

**Affiliations:** 1 Groundwater Environment Research Center, Geo-Environment Research Division, Korea Institute of Geoscience and Mineral Resources, Korea, Republic of; 2 Kyungpook National University College of Veterinary Medicine, Korea, Republic of; 3 AI-Bio Convergence Research Institute, Department of Industrial AI Engineering, Hoseo University, Korea, Republic of; 4 FM Animal Medical Center, Korea, Republic of; 5 Pet Industry, Daegu Haany University – Samseong Campus, Korea, Republic of; 6 Stellamed Co., Ltd., Korea, Republic of

**Keywords:** BCP, drinking water, *SMP*30 KO mouse, fecal microbiota, gut microbiome, AMPK, gut–liver axis, aging

## Abstract

Effective strategies are needed to increase the healthy lifespan and prevent age-related diseases in aging populations. Using *senescence marker protein* 30 knockout (*SMP*30 KO) mice—models that mimic human vitamin C (vitC) deficiency and exhibit accelerated aging—we investigated the effects of bioactive ceramic processed water (BCP) compared to natural mineral water (MW) and MW supplemented with vitamin C (MW-vitC) on gut microbial communities and hepatic metabolism. Due to pooled fecal sampling (n=1 composite library per group), microbiome results represent descriptive trends in diversity and composition. BCP was associated with discernible shifts in gut microbiota, including increased abundances of beneficial genera, such as *Akkermansia*, *Lactobacillus*, and *Allobaculum*, and the Muribaculaceae family. PICRUSt2 functional analysis suggested an enrichment in secondary metabolite biosynthesis, vitamin (e.g., retinol) metabolism, and xenobiotic biodegradation pathways. Furthermore, BCP was associated with significantly higher levels of activated hepatic AMP-activated protein kinase (AMPK), a key energy metabolism regulator, compared to control groups. Although microbiome findings are descriptive due to the study design, these results suggest BCP as a potential dietary intervention to help mitigate age-related metabolic decline and promote healthy ageing.

## Graphical abstract

**Figure:**
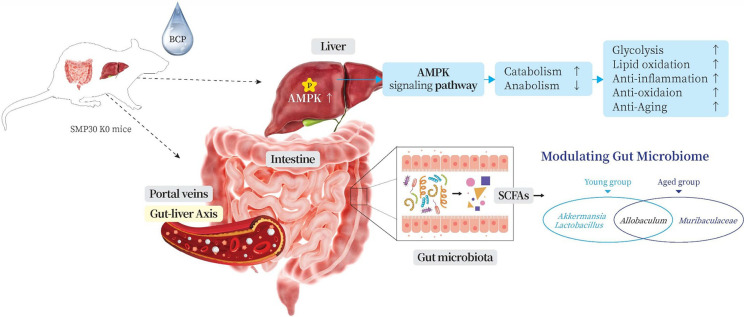
Effects of BCP on Gut Microbiome and Liver Metabolism in SMP30 KO Mice. Abbreviations: BCP, Bioactive ceramic processed water; SMP30 KO, Senescence marker protein-30 knockout; AMPK, Adenosine monophosphate (AMP)-activated protein kinase; SCFA, Short chain fatty acids.

## Social media summary

Our latest research presents a promising new approach to healthy aging. In a study using a premature aging mouse model, we discovered that bioactive ceramic-processed water (BCP) appears to improve both gut microbiota features and liver metabolism. Specifically, BCP promoted the growth of beneficial bacteria like *Akkermansia* and *Allobaculum*, which were linked to the activation of AMPK – a key metabolic regulator in the liver. This suggests that something as simple as drinking water can modulate the gut-liver axis to enhance metabolic health and potentially mitigate age-related diseases.

## Introduction

Currently, the proportion of the older population is rapidly increasing worldwide, posing significant challenges to public health and economic systems. In this social context, efforts to understand the biological mechanisms for healthy aging are becoming essential. Healthy lifespan extension and age-related disease prevention are the crucial research priorities in a rapidly aging society. Aging is not merely the passage of time but a complex biological process influenced by various genetic and environmental factors (López-Otín et al., 2023). Expression of the *senescence marker protein (SMP)30*, a key aging marker, decreases with age (Fujita et al., 1996; Maruyama et al., 2010). Notably, this decline is mechanistically linked to several hallmarks of aging, including loss of proteostasis and mitochondrial dysfunction (López-Otín et al., 2023). Being a multifaceted protein, *SMP30* is also involved in calcium homeostasis and vitC biosynthesis in most mammals, but not in humans (Drouin et al., 2011).

*SMP30* knockout (KO) mice, which cannot synthesize vitC, mimic human vitC deficiency and exhibit various premature aging-like phenotypes, including a shortened lifespan, increased oxidative stress, mitochondrial dysfunction, and enhanced inflammatory responses, compared to the wild-type mice (Maruyama et al., 2004; Ishigami, 2010; Park et al., 2010; Saga et al., 2021). Crucially, *SMP30*’s role extends beyond vitC synthesis; its deficiency exacerbates cellular damage by disrupting calcium homeostasis, rendering hepatocytes highly susceptible to apoptosis (Ishigami et al., 2002). Although not direct proxies of the multifactorial human aging process, these pathologies make *SMP30* KO mice vital animal models to study the mechanisms underlying age-associated metabolic decline and facilitate the development of suitable interventions for chronic vitC deficiency and cellular stress. Furthermore, *SMP30* KO mice exhibit prominent metabolic syndrome-like pathologies, such as impaired liver function, fatty liver, insulin resistance, and glucose metabolism disorders, suggesting that *SMP30* plays an essential role in maintaining liver homeostasis (Kondo and Ishigami, 2016).

Recent studies have revealed the significant impacts of the gut microbiota on the host’s health, particularly the host’s immune function, metabolic activity, and aging process (Gao et al., 2018; Best et al., 2025). Gut microbial dysbiosis (microbial balance disruption) is closely associated with various diseases and conditions, such as obesity, diabetes, inflammatory bowel disease, neurodegenerative diseases, and accelerated ageing (Sankararaman et al., 2023; Nie et al., 2025; Yan et al., 2025; Yu et al., 2025; Zhang et al., 2025). Consequently, modulation of the compositions and functions of gut microbial communities has gained attention as a novel therapeutic strategy for increased health span (Sun et al., 2020; Xiao et al., 2025).

Drinking water, an essential part of daily life, continuously interacts with the gut microbial environment, with the result that its type and composition significantly influence the structures and functions of gut microbial communities (Vanhaecke et al., 2022). Unlike nutritional supplements or pharmaceuticals, drinking water is the substance most easily and continuously consumed in daily life, thus holding high potential as a suitable vehicle for long-term health intervention studies. Drinking water source and solute composition have been associated with distinct gut microbiota signatures in population cohorts (Bowyer et al., 2020; Vanhaecke et al., 2022). However, research focusing on the combined impact of water’s mineral profile and its unique physicochemical structure on the gut microbiome is still lacking.

This study specifically focused on investigating the impacts of bioactive ceramic-processed water (BCP), a water type derived from natural groundwater sources and subsequently treated with ceramic composed of granite and pure red clay. This specialized post-processing distinguishes BCP from conventional natural mineral water (MW, groundwater-based), intentionally positioning it as a functional water designed to induce specific biological activity. The proprietary ceramic filtration system aims to optimize the mineral profile (e.g., elevated potassium and trace elements, including barium and gallium) and cause physicochemical modification. BCP is currently a novel test substance, not widely available or commercially standardized, and not yet commercially available as a health intervention, but holds high translational potential. Previous studies (Yun et al., 2004; Lee et al., 2005) have suggested that these unique elemental differences confer potential hepatoprotective properties in animal models. Although human clinical data are absent due to its novel status, and its effects on human health are yet to be determined, the constituent elements of BCP are based on generally recognized safe natural minerals. Crucially, promising pre-clinical findings indicating hepatoprotective properties (Yun et al., 2004; Lee et al., 2005) necessitate urgent mechanistic investigation into how its distinct physicochemical properties confer biological activity. We hypothesized that these elemental differences confer a unique biological activity capable of modulating the gut environment, thereby potentially impacting host metabolism. Given that *SMP30* KO mice exhibit severe age-related liver pathologies and metabolic dysfunction, BCP’s previously reported hepatoprotective potential provides a strong rationale for investigating its systemic effects, especially in the context of vitC deficiency and accelerated aging.

Considering BCP’s unique properties and the profound impact of the gut microbiota on host metabolism (particularly via the gut-liver axis), as well as the liver acting as a central metabolic hub, we explored the metabolic association between the gut and liver. Specifically, we assessed hepatic AMP-activated protein kinase (AMPK) activation, a crucial energy metabolism regulator, to clarify the potential systemic effects of BCP. The objective of this study was to evaluate the impacts of natural mineral water (MW), MW supplemented with vitC (MW-vitC), and BCP on the diversity, composition, and predicted metabolic functions of faecal microbiota in *SMP30* KO mice. MW-vitC was included as a critical physiological control to dissect the effects of BCP. As vitC supplementation has been previously shown to partially mitigate some *SMP30* deficiency phenotypes (Maruyama et al., 2004), this MW-vitC control is vital to ascertain whether the observed benefits of BCP are solely due to compensating for the vitC deficiency or arise from its distinct physicochemical properties and modulation of the gut–liver axis. Furthermore, we sought to assess the distinct influence of BCP on the gut microbial ecosystem and examine its potential to mitigate age-related pathologies and improve metabolic health.

## Material and methods

### Animals

*SMP30* KO mice were generated via gene targeting, as previously described (Ishigami et al., 2002). The mice were housed in a room at 22 ± 3 °C under 50 ± 10% relative humidity and a 12/12-h light-dark cycle. Food and water were provided *ad libitum.* Body weight was measured once a week throughout the experimental period. After each 8-week period for the young group and 10-week period for the aged group drinking water regimen, the livers of mice were rapidly excised and stored at –70 °C for further analyses, including AMPK activation assessment.

### Drinking water preparation

**Scheme 1.**
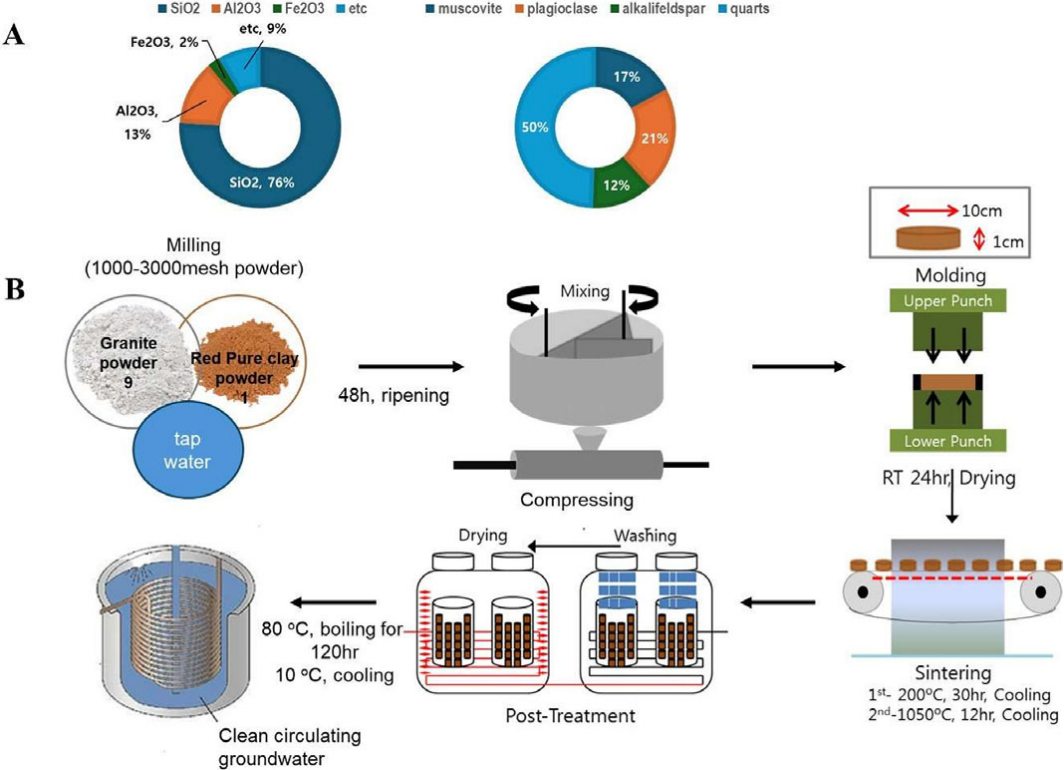
Schematics of bioactive ceramic processed water (BCP) preparation procedures. (A) Chemical compositions and mineral constituents of bioactive ceramic stone. (B) BCP manufacturing process.

Three distinct drinking water types were used in this study: Natural MW (groundwater resources), MW-vitC (L-ascorbic acid; 1.5 g/L; Sigma), and BCP. Scheme 1 outlines the bioactive ceramic stone and BCP production process. To create bioactive ceramic stone, granite and loess powders were mixed in a 9:1 ratio, matured for 48 h, compressed at 200 °C for 30 h, and sintered at 1,050 °C for 12 h. Subsequently, BCP was produced by heating the ceramic stone with MW at 80 °C for 120 h, followed by filtration through a 0.45-μm pore-size filter.

### Experimental design and sampling

**Figure 1.**
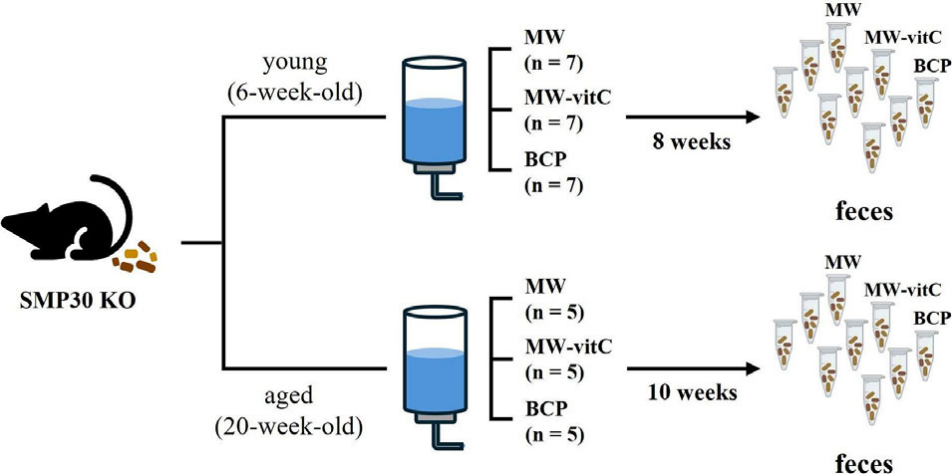
Experimental design. Faecal microbiota analyses of age-specific senescence marker protein (*SMP*)-*30* knockout (KO) mice (vitamin C-deficient) under different drinking water regimens.

Six- and 20-week-old male-specific pathogen-free *SMP30* KO mice were divided into three experimental groups for each age cohort. Young group (six-week-old) comprised seven mice per subgroup (*n* = 7), whereas the aged group (20-week-old) comprised five mice per subgroup (*n* = 5; Figure 1). All mice were fed a vitC-free diet throughout the 8–10-week drinking water regimen. *SMP30* KO mice depend on exogenous vitamin C. We therefore included MW-vitC as a physiological comparison to partially offset ascorbate deficiency, contrasted with MW and BCP. For subsequent microbiota analyses, fresh faecal samples were collected immediately before and after treatment. Individual faecal samples were collected, but for sequencing, samples were pooled within each group to generate a representative profile. The samples were placed in autoclaved Eppendorf tubes and stored at –80 °C until use.

### DNA extraction and *16S rRNA* gene sequencing

Genomic DNA was extracted from the faecal samples using the QIAamp DNA Stool Mini Kit (QIAGEN, USA), following the manufacturer’s instructions. DNA quantity was assessed using the Qubit fluorometer (Thermo-Fisher Scientific, USA), followed by storage at –20 °C. For microbiome profiling, individual faecal DNA extracts from mice within the same age × water type × timepoint cell were quantified and then normalized to 10 ng/μL. The normalized extracts were pooled at equal mass to generate one composite DNA sample per cell (Total *N* = 12 pooled libraries: 2 ages × 3 water types × 2 timepoints). Pooling was implemented to reduce per-sample sequencing variability and to obtain a cell-level composite profile representative of the group for descriptive comparisons. Consequently, all downstream microbiome analyses are reported as descriptive trends without group-level inferential statistics (e.g., PERMANOVA or differential abundance testing). The pooled DNA served as template to amplify the V4 hypervariable region of the *16S rRNA* gene using the 515-F and 806-R primer pairs, including Illumina adapters and a unique 12-bp barcode for each sample (Caporaso et al., 2011). Sequencing was performed by Macrogen (Seoul, Korea) on the Illumina MiSeq platform (Illumina, USA), according to the manufacturer’s protocol.

### Sequence processing

Sequence data were analysed using the Quantitative Insights Into Microbial Ecology 2 pipeline (Bolyen et al., 2019). Demultiplexing (DADA2 pipeline), quality filtering, taxonomy assignment (Silva database version 138), and phylogenetic tree creation using the q2-phylogeny plugin with the MAFFT and FastTree2 programs were performed to sequence the reads obtained from the faecal samples. Raw sequence data have been deposited into the National Center for Biotechnology Information Sequence Read Archive (project number: PRJNA1237931).

### Diversity and statistical analyses

Statistical analyses of Quantitative Insights Into Microbial Ecology 2 artifacts were conducted using the phyloseq package (v 1.50.0) in R (v.4.2.1) (McMurdie and Holmes, 2013; R Core Team, 2022). Amplicon sequence variants (ASVs) assigned to non-bacterial and unknown sequences and those with less than 10 reads per sample were excluded. To normalize the sequencing depth across all samples, the samples were rarefied to 5,000 reads.

Alpha- and beta-diversity analyses were conducted using the R packages phyloseq and vegan (McMurdie and Holmes, 2013; Oksanen et al., 2022). Alpha diversity indices were calculated using the estimate_richness function in phyloseq. Beta diversity was evaluated using the Bray–Curtis dissimilarities (Bray and Curtis, 1957) and visualized via principal coordinate analysis. Due to the pooled design (*n* = 1 per condition), statistical hypothesis tests for diversity metrics were not performed.

Phylogenetic investigation of communities by reconstruction of unobserved states 2 (PICRUSt2) was used to predict the functions and metabolic pathways based on the Kyoto Encyclopedia of Genes and Genomes (KEGG) database (Kanehisa et al., 2016; Yang et al., 2023). Because the analysis unit was a pooled sample, microbiome outcomes are presented descriptively using relative abundance changes and heatmaps.

### Immunoblotting analysis

Liver pieces were lysed using the radioimmunoprecipitation assay buffer containing 0.1 mM/L Na_3_VO_4_ and protease inhibitor cocktail tablets (Roche, Mannheim, Germany). The lysate was centrifuged at 14,000 rpm for 20 min at 4 °C to obtain soluble proteins. Equal quantities (40 μg) of total protein were separated via sodium dodecyl sulphate–polyacrylamide gel electrophoresis and electro-transferred to polyvinylidene difluoride membranes (Millipore Co., Billerica, MA, USA). After blocking with 3% bovine serum albumin in Tris-buffered saline containing 2% Tween-20 (TBST), the membranes were incubated with appropriate dilutions of primary antibodies in 3% bovine serum albumin in TBST. The following primary antibodies were used: Anti-phospho-AMPKα and anti-AMPKα (Cell Signalling Technology Inc., Danvers, MA, USA) antibodies. After washing thrice with TBST, the membranes were incubated with the horseradish peroxidase-conjugated secondary antibodies. Specific binding was detected using the SuperSignal West Dura Extended Duration Substrate (Pierce, Rockford, IL, USA) and developed using a medical X-ray film (Kodak, Tokyo, Japan). Images of the protein bands were scanned, and band intensities were quantified using the ImageJ software. Statistical comparisons for Western blot data (performed on individual biological replicates, *n*=3 per group) were conducted using one-way ANOVA followed by Tukey’s post hoc test. A *p*-value < 0.05 was considered statistically significant.

### Results

#### Biometric measurements

**Figure 2.**
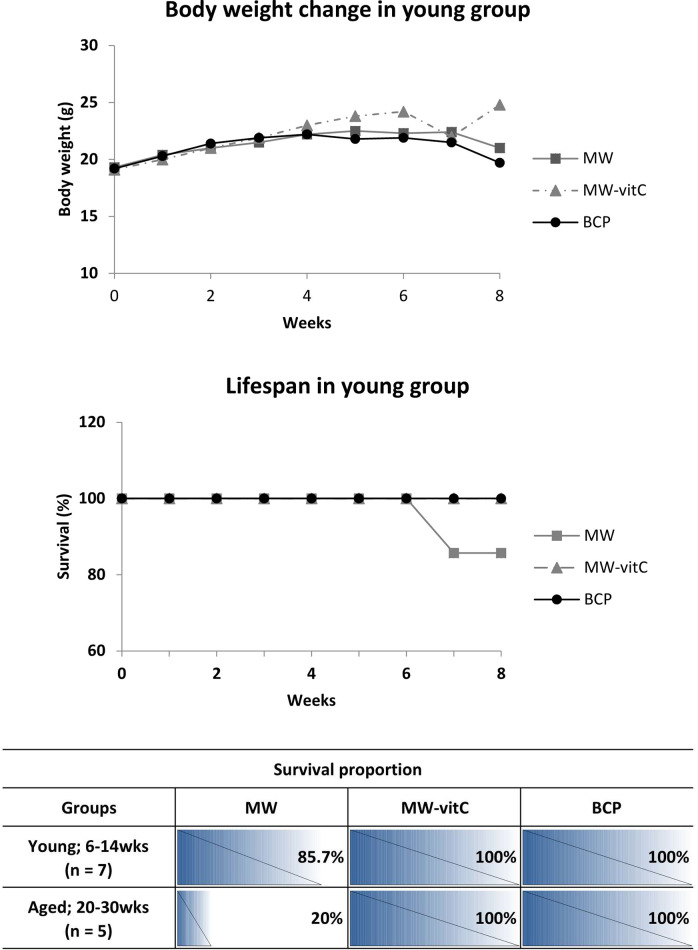
Biometric trends and lifespan data (*p* > 0.05; one-way analysis of variance [ANOVA]).

Body weight and survival were monitored weekly throughout the experimental period (Figure 2).

Concerning body weight changes, we observed that *SMP30* KO mice in young group fed MW-vitC and BCP gradually gained weight in the initial weeks (weeks 0–4), with no significant difference in early weight gain between these two groups. In contrast, the MW-treated group maintained their body weight for up to approximately week 5, after which they began to show a sharp weight loss towards the end of the experimental period. Interestingly, while the BCP-treated group also showed initial weight gain, their weight began to plateau or slightly decrease after approximately week 4, diverging from the continued steady gain observed in the MW-vitC group and showing a relatively stable, or slightly decreasing pattern of body weight towards the end of the experimental period.

As for lifespan, all mice in the MW-vitC and BCP-treated groups, across both young (6–14 weeks) and aged (20–30 weeks) subgroups, exhibited complete survival throughout the experimental period, maintaining 100% survival rate (Figure 2). However, in the MW-treated group, a decrease in survival was observed. Specifically, one mouse from the young group (initial *n* = 7) died naturally after 6 weeks, resulting in a survival rate of 85.7% (Figure 2). Furthermore, the aged subgroup of the MW-treated group (initial *n* = 5) exhibited a much lower survival rate of only 20%, indicating that four mice in this group died naturally after week 6, based on the overall observed decline in MW group survival (as detailed in the table).

This finding suggests that BCP has a positive effect on both body weight maintenance and survival rate in *SMP30* KO mice. Notably, it exhibits benefits comparable to vitamin C supplementation, particularly in improving survival and mitigating the severe weight loss observed in the MW group, although the sustained weight gain seen with MW-vitC is not fully replicated.

#### Microbial community diversity in response to water treatment and age

Next, *16S rRNA* gene sequencing of pooled 12 samples yielded 220,151 quality-filtered raw reads and 346 distinct bacterial ASVs upon further filtering and rarefaction.

**Figure 3.**
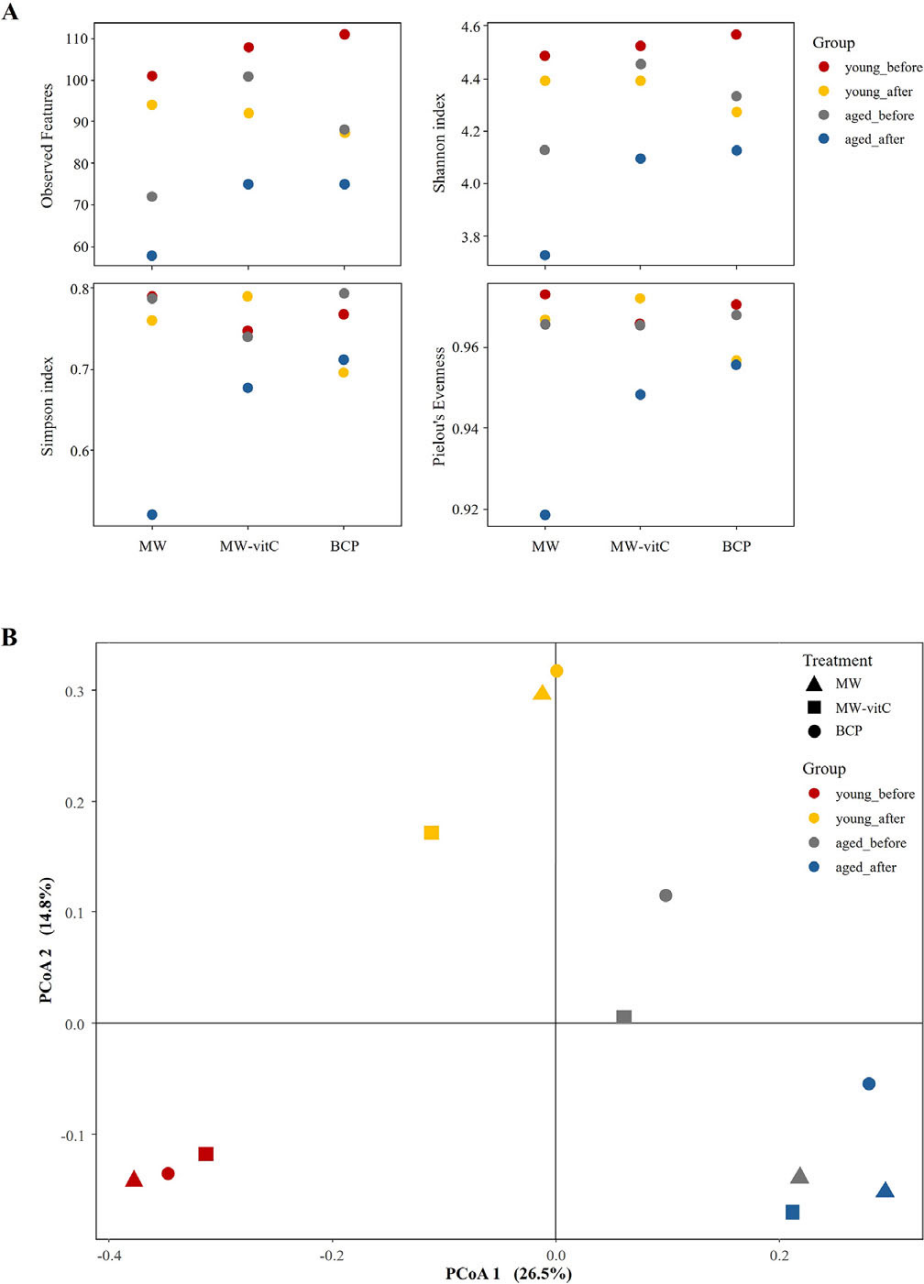
Faecal microbiota diversity. (A) Alpha diversity indices (observed features, Shannon, Simpson, and Pielou’s evenness indices) illustrating drinking water treatment and age-related patterns. (B) Principal coordinate analysis (PCoA) plot of Bray-Curtis dissimilarities by age and water type.

Alpha diversity assessment (Figure 3A; Supplementary Table 2) suggested lower microbial species richness (observed features and ASV richness) and evenness (Shannon index) in the post-treatment groups than in the pre-treatment controls. Notably, the MW group showed the lowest species richness compared to MW-vitC and BCP treated groups. Young mice (6-week-old) consistently maintained higher ASV richness and Shannon indices than the aged mice (20-week-old). Overall composition appeared similar across water types post-treatment.

Beta diversity analysis via principal coordinate analysis based on Bray–Curtis dissimilarities (Figure 3B) revealed apparent shifts in the community structures of the groups before and after drinking water treatment. Distinct compositional differences were also observed before and after water treatment in both the young and aged mice. However, overall composition appeared similar across water type treatments (e.g., MW vs. MW-vitC vs. BCP post-treatment).

#### Faecal microbiota composition changes based on the drinking water type and age

**Figure 4.**
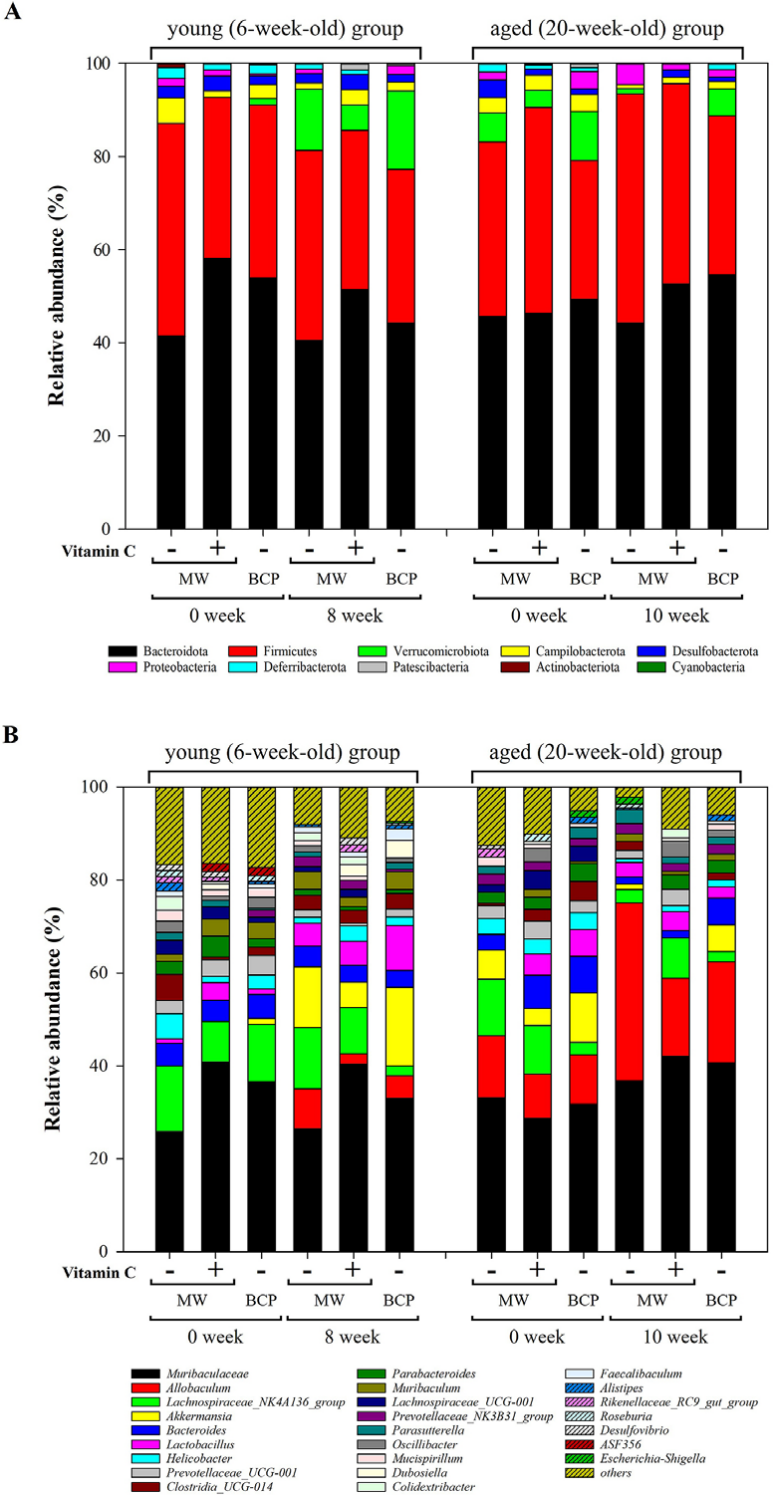
Relative abundances of key bacterial taxa in the faecal samples. (A) Phylum-level composition showing the relative frequencies across treatment groups and time points. (B) Genus-level composition indicating the top 25 most abundant genera.

Taxonomic analysis of faecal bacteria at the phylum and genus levels (Figure 4) revealed distinct shifts influenced by drinking water treatment and mouse age. Ten phyla were detected across all samples. Bacteroidota (48.53 ± 5.40%) and Firmicutes (38.61 ± 5.69%) were the most abundant, followed by Verrucomicrobiota (5.36 ± 5.36%), Campylobacterota (2.52 ± 1.28%), and Desulfobacterota (1.96 ± 1.03%; Figure 4A). Notably, Firmicutes/Bacteroidota (F/B) ratios were increased in both the young and aged mice after all drinking water treatments (Supplementary Figure 1).

Changes in the dominant phyla, Bacteroidota and Firmicutes, differed with age. In young mice, abundances of both phyla were generally decreased after all water treatments. Specifically, the decrease in Bacteroidota abundance was more pronounced in the MW-vitC and BCP groups, with reductions of 6.70 and 9.68%, respectively, compared to the 0.96% reduction in the MW group. MW and BCP groups exhibited Firmicutes abundance reductions of 4.86 and 4.22%, respectively, whereas the MW-vitC group showed only a 0.36% reduction. Conversely, the aged group exhibited slightly elevated abundances of both Bacteroidota and Firmicutes. Although MW group showed a 1.46% decrease in Bacteroidota abundance, the MW-vitC and BCP groups showed increased abundances of 6.32 and 5.18%, respectively. Firmicutes abundance increased by 11.76 and 4.46% in the MW and BCP groups, respectively, and decreased by 1.22% in the MW-vitC group.

Verrucomicrobiota exhibited significant age-dependent changes in relative abundance. Young groups exhibited an average of 11.37 ± 4.32% increase (MW: 13.14% increase; MW-vitC: 5.42% increase; BCP: 15.56% increase), whereas the aged groups showed an average of 4.52 ± 0.60% decrease (MW: decreased by 5.04%; MW-vitC: decreased by 3.68%; BCP: decreased by 4.84%) in Verrucomicrobiota abundance after all different drinking water treatments. Unlike those of Verrucomicrobiota, response patterns of other bacterial phyla varied without a consistent trend and did not show any significant changes based on the water type and age.

Campylobacterota abundance generally decreased in both the young (by 4.08%) and aged (by 2.44%) groups after MW treatment. However, MW-vitC uniquely increased the Campylobacterota abundance in the young group (by 1.88%) and decreased it in the aged group (by 1.88%). Notably, BCP consistently reduced the Campylobacterota abundance in both the young (by 1.14%) and aged (by 2.02%) groups, suggesting a common impact across age groups. (Note: these descriptions refer to changes in the pooled population average).

Responses of Proteobacteria also varied depending on the water type and age. MW increased the Proteobacteria abundance in the aged group (by 2.84%); however, the abundance remained stable or showed minimal changes in the young group. In contrast, MW-vitC decreased the Proteobacteria abundance in the young group (by 1.28%) but increased it in the aged group (by 1.38%). Interestingly, BCP increased the Proteobacteria abundance in the young group (by 1.52%) but significantly decreased it in the aged group (by 2.20%), indicating an age-specific effect different from that observed in the other treatments.

Desulfobacterota abundance consistently decreased in both the young (by 0.52%) and aged (by 3.78%) groups following MW treatment. Deferribacteriota abundance also decreased by 1.18% in the young group and 1.88% in the aged group after MW treatment, and 1.98% in the young group after BCP treatment. Patescibacteria abundance increased only in the young MW-vit C treatment group (by 1.40%). Notably, no other detected phyla exhibited significant changes based on the drinking water type and age.

Genus-level analyses revealed significant shifts influenced by water exposure and age (Figure 4B; Table 1). Abundances of *Akkermansia*, *Allobaculum*, and *Lactobacillus* were markedly increased and that of *Dubosiella* was increased in the young mice. However, abundances of genera, such as *Lachnospira*, *Prevotellaceae_UCG_001*, and *Parabacteroides*, were decreased in these mice. Abundances of *Allobaculum* and unclassified *Muribaculaceae* were significantly increased, whereas those of *Akkermansia*, *Lachnospiraceae_NK4A136*, *Bacteroides*, *Lachnospiraceae_UCG*, and *Helicobacter* were decreased in the aged mice. Notably, *Allobaculum* abundance was elevated in both the young and aged mice.

**Table 1.** Relative abundances of the top 25 faecal microbiota genera (% of total)

Genus	Young group			Aged group		
	MW	MW-vitC	BCP	MW	MW-vitC	BCP
Unclassified *Muribaculaceae*	0.66	–0.52	–3.54	3.62	13.28	8.90
*Allobaculum*	8.56	2.26	4.80	24.98	7.38	11.16
*Lachnospiraceae_NK4A136*	–1.02	1.22	–10.16	–9.32	–1.82	–0.50
*Akkermansia*	13.14	5.42	15.56	–5.04	–3.68	–4.84
*Bacteroides*	–0.36	–0.90	–1.46	–1.82	–5.50	–2.16
*Lactobacillus*	3.84	1.44	8.46	2.90	–0.58	–3.44
*Helicobacter*	–4.08	1.88	–1.14	–2.44	–1.88	–2.02
*Prevotellaceae_UCG–001*	–1.30	–2.94	–2.54	–1.00	–0.34	–2.52
*Clostridia_UCG–014*	–2.40	2.24	1.58	1.42	–2.52	–2.84
*Parabacteroides*	–1.52	–3.78	–1.02	–2.36	0.42	–1.06
*Muribaculum*	2.26	–1.62	0.36	1.60	–0.96	0.96
*Lachnospiraceae_UCG–001*	–1.96	–0.88	–1.06	–1.66	–3.96	–3.36
*Prevotellaceae_NK3B31*	2.18	1.76	–1.16	–0.04	–0.18	0.50
*Parasutterella*	–0.68	–1.28	1.14	1.30	1.38	–0.82
*Oscillibacter*	–1.10	–0.88	–1.34	0.00	0.52	1.44
*Mucispirillum*	–1.18	–0.46	–1.98	–1.88	–0.88	0.44
*Dubosiella*	0.00	1.26	3.76	0.00	0.14	0.68
*Colidextribacter*	–1.32	1.00	0.00	0.00	1.88	0.00
*Faecalibaculum*	0.10	1.14	1.66	0.00	0.00	0.00
*Alistipes*	–1.36	0.00	0.12	0.38	0.00	–0.10
*Rikenellaceae_RC9*	–1.24	0.64	0.00	–1.84	0.00	0.00
*Roseburia*	–1.30	0.00	–0.78	0.80	–1.58	0.00
*Desulfovibrio*	–1.28	0.24	0.00	–0.74	0.00	0.00
*ASF356*	0.00	–1.76	–1.74	0.00	0.00	0.00
*Escherichia-Shigella*	0.00	0.00	0.38	1.54	0.00	–1.38

Values are *Δ* in percentage points (pp); positive means increase, negative means decrease.

#### Differential effects of the drinking water type and age on the faecal microbiota

**Figure 5.**
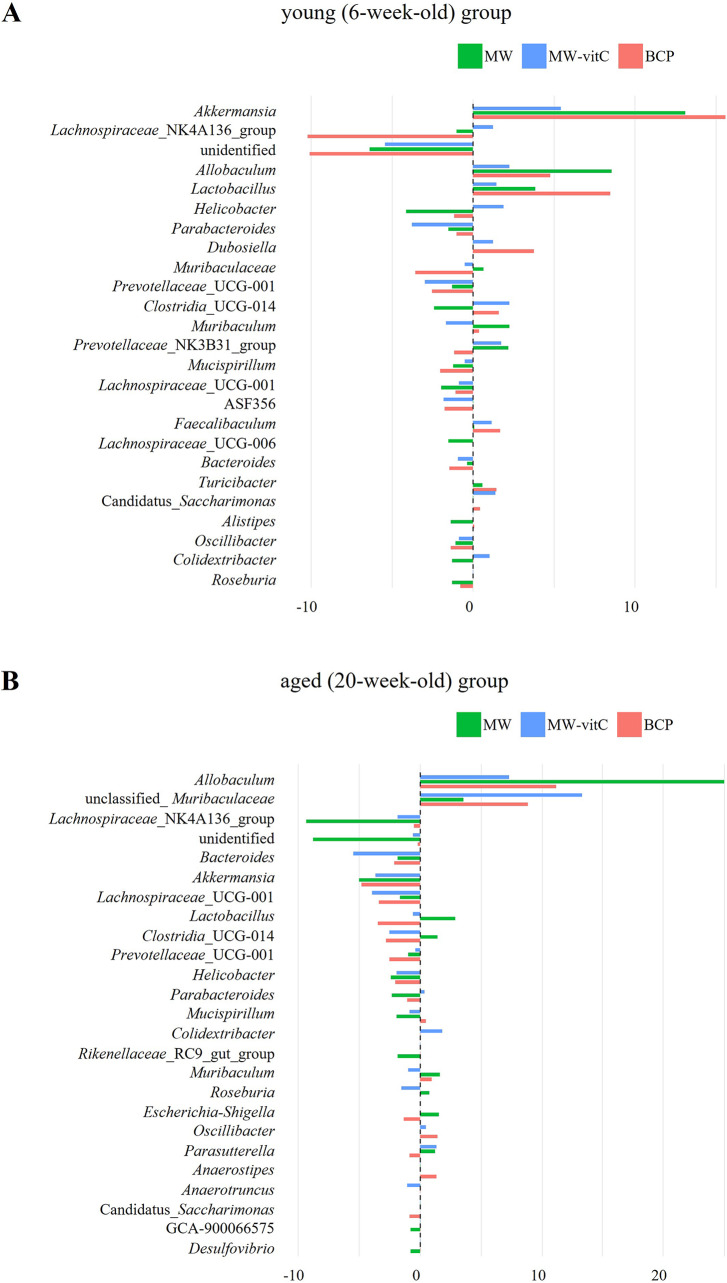
Genus-level differences in relative abundance are shown separately by young (6-week-old; A) and aged (20-week-old; B) SMP30-KO mice across drinking-water regimens. Each bar depicts the within-cell change for one pooled library; the dashed vertical line indicates no change.

Genus-level differences in relative abundance indicated that faecal microbial communities shifted in a water regimen and age-dependent manner (Figure 5). In the young (6-week-old) group (Figure 5A), *Akkermansia* showed a clear positive delta across all regimens, most prominently with BCP. *Allobaculum* and *Lactobacillus* showed modest positive changes. By contrast, Lachnospiraceae_NK4A136_group exhibited the largest decreases in BCP, while several genera showed small or near-zero deltas, indicating non-uniform responses across taxa.

In the aged (20-week-old) group (Figure 5B), patterns differed from those in the young mice. *Allobaculum* showed pronounced positive delta in all regimens, accompanied by increases in unclassified_*Muribaculaceae*, whereas *Akkermansia* and *Bacteroides* tended to show negative delta.

The genus-level heatmap likewise showed heterogeneous, regimen and age-specific changes (Supplementary Figure 2). *Allobaculum* retained an increase in both the young and aged groups, with varied changes depending on water type. Main differences between the age was *Akkermansia*, which increased in the young group with an average 11.4% and compare than pre-treatment, while decreased with 4.5% in the aged group. In contrast, unclassified_*Muribaculaceae* was increased in the aged group (average 8.6%) and decreased in the young group (average 1.1%) at post treatment. Collectively, these observations suggest a reorganization of the community that varies by water type and age, rather than a single directional shift affecting all taxa.

#### Predicted metabolic functions of the faecal microbiota

**Figure 6.**
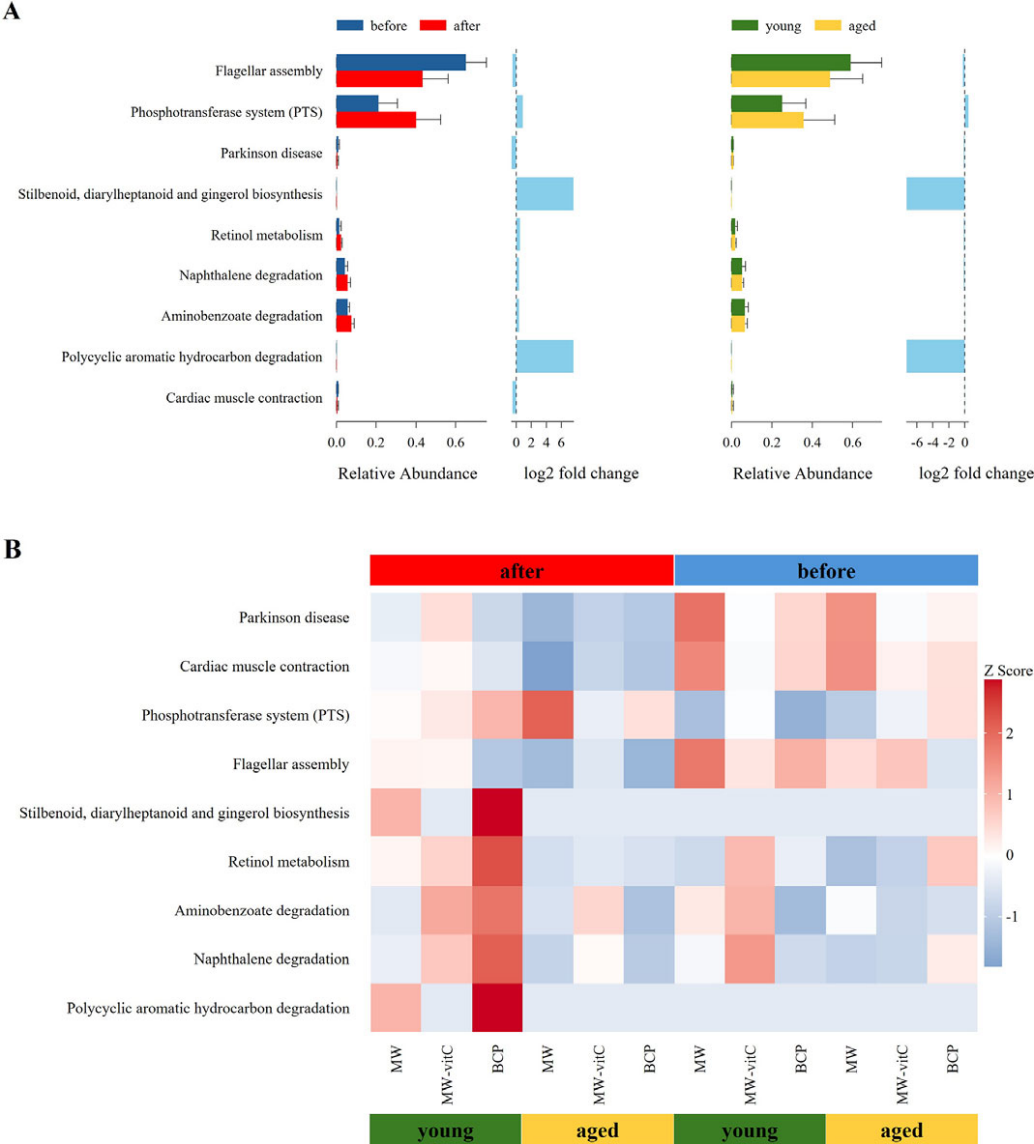
Predicted metabolic functions of the faecal microbiota. (A) Differences in the abundances of the annotated Kyoto Encyclopaedia of Genes and Genomes (KEGG) level three pathways stratified by drinking water type and age. (B) Heatmap showing the abundances of different KEGG pathways across sample groups.

Metabolic profile of the faecal microbiota, predicted using *16S rRNA* genes with the PICRUSt2 pipeline and KEGG database, revealed differential responses to drinking water type and age (Figure 6). Nine level 3 KEGG pathways were found to be differentially abundant across the groups.

Across all groups, flagellar assembly pathway was notably enriched in the pre-treatment young mouse groups (Figure 6A). However, the phosphotransferase system was predominant in the post-treatment aged mouse groups (Figure 6A).

Critically, the most apparent changes in metabolic pathway profiles were observed in the young mice of the BCP group. These included a distinct upregulation in various secondary metabolite biosynthesis pathways (stilbenoid, diarylheptanoid, and gingerol biosynthesis), cofactor and vitamin metabolism (retinol metabolism), and xenobiotic biodegradation and metabolism pathways. Although the biosynthetic and degradative metabolism pathways appeared higher, those essential for core metabolic functions were downregulated in the MW groups. Notably, MW-vitC groups showed no clear pattern of metabolic pathway changes.

#### AMPK activation in response to BCP administration

We investigated the association between drinking water type and hepatic metabolism by assessing the activation of AMPK, a crucial metabolic energy regulator, in the liver.

**Figure 7.**
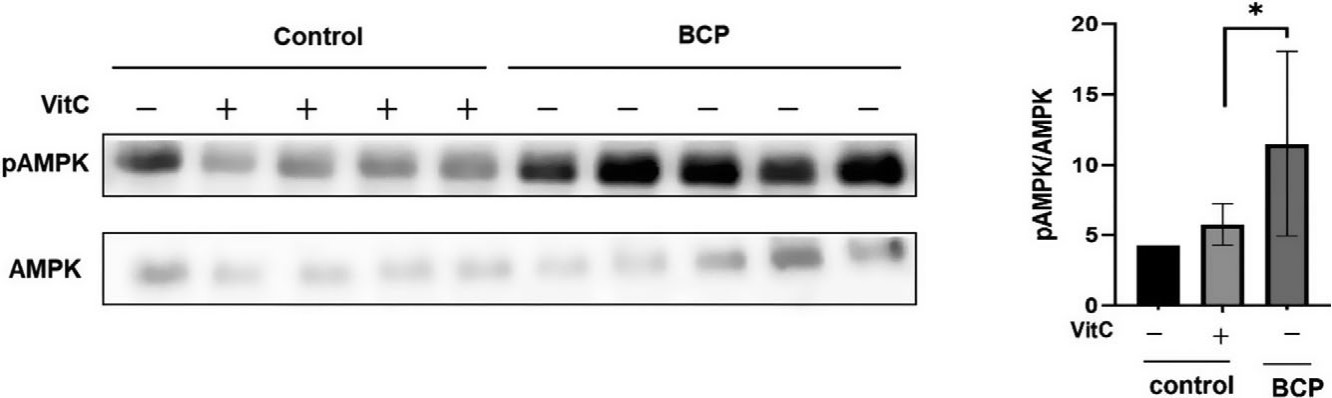
Phospho- and total AMP-activated protein kinase (AMPK) levels in the liver. (A) Immunoblotting analysis of the phospho- and total AMPK levels. (B) Quantitative analysis of band intensities in (A) using the National Institutes of Health (NIH) ImageJ software. Values are expressed as the mean ± standard deviation (SD). *p < 0.05*. SMP30* KO mouse experimental groups were as follows: Negative control (MW), positive control (MW-vitC), and treatment (BCP) groups.

Notably, BCP significantly increased AMPK activation in the liver tissues of the treated mice (Figure 7). Quantitative immunoblotting analysis revealed significantly higher phospho-AMPK levels than total AMPK levels in the BCP group (Figure 7A and B). This effect was statistically significant (*p* < 0.05) compared to the MW and MW-vitC control groups based on biological replicates (*n*=3).

### Discussion

In this study, we used the *SMP30* KO model mice to assess the mechanisms by which different drinking water types (natural MW, MW-vitC, and BCP) affect the gut microbial communities and their predicted metabolic functions. Notably, the tested water interventions consistently altered the gut microbial diversity. BCP uniquely influenced the microbial composition and metabolic pathways, causing beneficial shifts, particularly by promoting health-associated bacterial and metabolic activities. We identified a crucial link between these microbial changes and activation of hepatic AMPK, a key metabolic regulatory enzyme, providing insights into the health-promoting mechanisms of BCP.

Alpha diversity analysis (Figure 3A; Supplementary Table 2) revealed obvious reductions in microbial richness (observed features) and diversity (Shannon index) across all treatment groups, with the MW group showing the lowest diversity. vitC deficiency possibly inherently contributed to this reduction in gut microbial diversity in *SMP30* KO mice, consistent with previous reports on various vitC-deficient models (Otten et al., 2021; Pham et al., 2021a; Shneyderman et al., 2025). However, the less pronounced decrease in diversity in the MW-vitC and BCP groups suggests that specific components (e.g., exogenous vitC) or inherent properties (e.g., unique mineral composition or structural characteristics) of drinking water partially mitigate this reduction. This provides initial evidence of the stabilizing effects of BCP on the gut microbial environment. Despite changes in richness and Shannon diversity, Simpson’s index, and Pielou’s evenness remained consistent across treatments, indicating that, although the total number of bacterial types decreased, their relative abundances were maintained. Notably, selective enrichment of specific taxa (e.g., *Akkermansia*, *Lactobacillus*, and *Allobaculum*) can coincide with lower alpha diversity due to a dominance effect in compositional data (Fernandes et al., 2014). Thus, diversity indices and taxon level changes need not move in the same direction in our dataset (Gloor et al., 2017; Zmora et al., 2018; Stojanov et al., 2020). Taken together, this perspective explains why water-driven increases of particular genera can coexist with reduced overall diversity. The marked increase in distance between the pre- and post-treatment samples in the beta diversity analysis (Figure 3B) clearly implied that exposure to treated water, rather than the water type itself, drives bacterial community shifts. These findings collectively suggest that even regular water consumption impacts the faecal microbiota across all ages. Age played a significant role in this study, with young mice (6-week-old) exhibiting higher ASV richness and Shannon indices than aged mice (20-week-old). This aligns with the established patterns of age-related gut microbial alterations, where diversity typically declines with age, often leading to increased dysbiosis susceptibility (Escudero-Bautista et al., 2024; Gyriki et al., 2025). Overall, these observations indicate a regimen- and age-dependent reorganization of the gut community rather than a uniform shift across all taxa. In particular, selective enrichment of particular genera can coincide with lower alpha diversity, so diversity indices and taxon-level changes need not move in the same direction. Our 16S rRNA sequencing analysis revealed distinct shifts in faecal bacterial composition at the phylum and genus levels influenced by both the drinking water type and mouse age (Figure 4). Bacteroidota and Firmicutes were the most abundant phyla in the samples, confirming their prominence in the healthy mammalian gut (Jandhyala et al., 2015; Rinninella et al., 2019; Koh et al., 2023). F/B ratio was increased in both the young and aged mice after drinking water treatment (Supplementary Figure 1). Elevated F/B ratio is often associated with dysbiosis in various metabolic conditions, including obesity and metabolic syndrome (Stojanov et al., 2020). These associations and the inherent vitC deficiency in our *SMP30* KO model suggest that interpretation of F/B ratio changes requires careful consideration and comparative analysis with normal wild-type groups for conclusive findings.

Notably, we observed age-dependent changes in the abundance of Verrucomicrobiota phylum, primarily comprising the *Akkermansia* genus. Verrucomicrobiota abundance was increased by an average of 11.37% in the young mice but decreased by 4.52% in the aged mice. This divergent response highlights the age-specific effects of water intervention on this phylum. Interestingly, BCP uniquely influenced the Proteobacteria responses by increasing the Proteobacteria abundance in the young group and decreasing it in the aged group (2.20%). Increased Proteobacteria abundance is often considered a signature of dysbiosis and inflammation (Litvak et al., 2017), suggesting that BCP exerts anti-inflammatory and dysbiosis-correcting effects in aged mice (Parker et al., 2020; Fu et al., 2024).

Genus-level analysis (Figure 4B; Table 1) further revealed apparent water-dependent changes with varying prevalence across age groups. Abundances of beneficial genera, such as *Akkermansia*, *Allobaculum*, and *Lactobacillus*, were increased in the young mice, whereas those of *Allobaculum* and unclassified *Muribaculaceae* were obviously increased in the aged mice. Interestingly, *Allobaculum* abundance was increased in both the young and aged mice, indicating a consistent positive outcome across all water treatment groups.

BCP notably promoted the abundance of key beneficial genera, exerting age-specific effects. Relative abundances of *Akkermansia*, *Lactobacillus*, and *Allobaculum* were particularly high in young mice administered BCP. *Akkermansia muciniphila*, a prominent member of Verrucomicrobiota, is a next-generation probiotic using mucin in the gut. It produces short-chain fatty acids (SCFAs), such as acetate and propionate, which are vital nutrients for other gut bacteria and the host (Rodrigues et al., 2022; Wang et al., 2022; Aja et al., 2025; Liu et al., 2025). This bacterium enhances the gut barrier function, reduces inflammation, and positively impacts various metabolic and systemic disorders, including obesity, diabetes, and neurological disorders, despite showing a tendency to decline with age (Pei et al., 2023; Zeng et al., 2023; Zheng et al., 2023; Gofron et al., 2024; Zhao et al., 2024; Ma et al., 2025). *Allobaculum*, a bacterium in the Firmicutes phylum, also degrades mucin, performing functions similar to *A. muciniphila* in maintaining the intestinal mucus layer health and producing beneficial SCFAs, such as butyrate (van Muijlwijk et al., 2021; Kalkan et al., 2025). *Lactobacillus*, a well-known probiotic (Firmicutes phylum), plays a crucial role in the development of early life microbial communities and the immune system via interactions with mucosal immunity and competition with pathogens (Heczko et al., 2024). In the aged group administered BCP, unclassified *Muribaculaceae* was the next most abundant genus after *Allobaculum. Muribaculaceae* family (Bacteroidetes phylum) produces SCFAs primarily from endogenous (mucin glycans) and exogenous (dietary fibre) polysaccharides (Zhu et al., 2024) and plays a crucial role in the gut microbial ecosystem via cross-feeding relationships with other probiotics, such as *Bifidobacterium* and *Lactobacillus* (Zhu et al., 2024). These findings suggest that BCP fosters an environment conducive to the growth of health-promoting bacteria, thereby improving gut integrity, reducing inflammation, and enhancing overall metabolic function (Lin et al., 2025).

Our observation that BCP altered gut microbial diversity and composition, with increases in genera such as *Akkermansia*, unclassified *Muribaculaceae*, and *Allobaculum*, is consistent with the broader notion that water characteristics can remodel gut microbial communities (Bowyer et al., 2020; Vanhaecke et al., 2022). Our study specifically highlights BCP, which differs from MW not only in its processing but also in its physicochemical profile. As shown in Supplementary Table S1, BCP contains higher concentrations of potassium (K), barium (Ba), and gallium (Ga) compared to the MW control. While these trace elements remained within safe drinking water limits (e.g., Barium < 700 ppb) of the WHO guideline, it is possible that these specific elemental differences contributed to the observed biological shifts. Future studies should aim to decouple the effects of the mineral content from the structural properties of the water.

Genus-level differences indicate a regimen and age-dependent reorganization of the gut community rather than a uniform shift affecting all taxa (Figure 5, Supplementary Figure 2). *Allobaculum* showed a promoted increase across water regimens and age, especially MW treatment. Consistent with a previous report (Jian et al., 2023) (Figure 4B) *Akkermansia* abundance notably increased in the young mice, particularly following BCP treatment, reinforcing its role as a key responder to BCP. Notably, increases in *Akkermansia* and *Lactobacillus* are often interpreted as a favourable signal for supporting gut stability, such as barrier maintenance, acidification, and cross-feeding (Plovier et al., 2017; Gao et al., 2019; Rodrigues et al., 2022; Heczko et al., 2024; Zhu et al., 2024).

Metabolic profile prediction using PICRUSt2 (Figure 6) revealed functional alterations depending on the drinking water type and age. Generally, flagellar assembly pathways were enriched before water treatment in young mice, indicating high microbial motility, host adhesion, and biofilm formation, which are crucial for initial gut colonization and niche establishment (Colin et al., 2021). Conversely, the phosphotransferase system was predominant after water treatment in aged mice, indicating enhanced sugar uptake capacity and metabolism. These findings indicated increased carbohydrate utilization flexibility in the microbial community, effectively adapting to altered nutrient availability and host health conditions (Deutscher et al., 2006).

Notably, the most distinct changes in metabolic pathway profiles were observed in the young mice of the BCP group. These included the marked upregulation of various secondary metabolite biosynthesis (stilbenoid, diarylheptanoid, and gingerol biosynthesis), cofactor and vitamin metabolism (retinol metabolism), and xenobiotic biodegradation and metabolism pathways. Stilbenoids, diarylheptanoids, and gingerols are plant-derived phytoalexins exhibiting natural antioxidant, anti-inflammatory, and anti-cancer activities (He et al., 2024; Abdullah et al., 2025; Meng et al., 2025). This suggests that the activated biosynthetic pathways increased the production of host health-promoting metabolites by the gut microbiota. Increased vitamin (retinol) metabolism was particularly noteworthy in vitC-deficient *SMP30* KO mice. Although vitamins A and C exhibit distinct metabolic and absorption mechanisms, both are crucial for antioxidant activity, immune functions, and overall gut health (Pham et al., 2021a, 2021b). The observed increase in retinol metabolism, along with the known beneficial modulation of the gut microbiome by vitamin C (Pham et al., 2021b), suggests plausible indirect interactions or a synergistic link between vitamins A and C, potentially mediated by the gut microbiota, which alleviates the pathologies arising from vitamin deficiency. Moreover, activation of xenobiotic biodegradation pathways by the gut microbiota has the potential to contribute to host defense against diverse foreign compounds by enhancing detoxification (Collins and Patterson, 2020). In contrast, although the MW groups showed the upregulation of both the biosynthetic and degradative metabolism pathways, this was accompanied by a downregulation of the pathways critical for fundamental host health and survival (e.g., core metabolic processes essential for cellular maintenance). Therefore, despite some metabolic activity enhancement, the overall beneficial impact on the host’s well-being was compromised in these groups. MW-vitC groups showed no significant metabolic pathway changes, indicating limited impact. Collectively, these distinct outcomes highlight the unique and targeted metabolic improvement effects of BCP that are particularly beneficial to mitigate age-related metabolic decline and extend far beyond simple vitC supplementation.

Liver, the central systemic energy metabolism and detoxification organ, undergoes various functional and structural changes, including increased oxidative stress, mitochondrial dysfunction, inflammation, and hepatic fibrosis, with age, all of which increase the liver disease risk (Friedman, 2003). AMPK is a crucial cellular energy sensor; cellular energy depletion activates AMPK to promote catabolism (energy production) and suppress anabolism (energy consumption), thereby restoring the energy balance (Shirwany and Zou, 2014). In the liver, AMPK plays a complex role as a key regulator of lipid and glucose metabolism, the central component of antioxidant and anti-inflammatory defence, and a significant modulator of cell growth and survival (Jeon, 2016; Rey and Tamargo-Gómez, 2023). Under oxidative stress conditions, AMPK activates sirtuin 1, which stimulates the forkhead box O1 transcription factor, enhancing the mitochondrial functions and mitigating oxidative stress-induced cellular injury (Guan et al., 2025). Therefore, AMPK functions as a core defence system counteracting age-related liver decline, maintaining energy homeostasis, and minimizing cellular damage (Salminen and Kaarniranta, 2012; Burkewitz et al., 2014; Garcia and Shaw, 2017). Although AMPK responsiveness to stress in the liver weakens with age, its multifaceted functions offer significant therapeutic potential in delaying the progression of various metabolic liver diseases and conditions, such as fatty liver, diabetes, obesity, liver injury, impaired regeneration, and other age-related conditions (Herzig and Shaw, 2018).

After determining the positive impact of BCP on the gut microbiome, we evaluated its effects on liver tissues by assessing AMPK activation. *SMP30* KO mice in BCP groups exhibited significantly higher activated AMPK levels than those in the MW and MW-vitC control groups (Figure 7). Although additional experiments are necessary to determine whether BCP can directly activate AMPK in the liver, our study suggests that it indirectly influences AMPK activation via the gut microbiome. Specifically, BCP modulates the gut microbial composition, promotes the growth of specific beneficial bacteria, and increases the production of microbial metabolites (e.g., SCFAs and secondary metabolites identified via PICRUSt2 analysis), which facilitate liver AMPK activation via the gut–liver axis (Ryu et al., 2020; Li et al., 2025; Wang et al., 2025). This suggests a fascinating interplay where BCP modulates the gut environment, leading to systemic metabolic benefits. BCP possibly exerts systemic effects beyond localized gut health improvement, impacting crucial organ functions, such as liver metabolism.

In this study, BCP exerted unique and beneficial effects on the gut microbiota and their metabolic functions in *SMP30* KO mice, particularly young mice. Importantly, these effects were associated with increased liver AMPK activation. Our findings suggest BCP as a promising potential dietary intervention for the prevention and management of age-related metabolic diseases.

Future studies should investigate the specific active components and physical structure of BCP to elucidate the molecular mechanisms underlying its effects on gut microbiota–host interactions and AMPK activation.

### Limitations

This study has several limitations that must be considered. First and foremost, to obtain sufficient DNA yield and reduce sequencing variability, faecal samples were pooled within each group (*n*=1 composite library per cell). This design precludes the estimation of biological variance and prevents the use of group-level inferential statistics (e.g., PERMANOVA (Anderson, 2001), LEfSe) for the microbiome data; thus, all microbiome findings reported here are descriptive and hypothesis-generating. Additionally, while survival trends were observed, the small sample size in the aged cohort (*n*=5) limits the statistical robustness of the lifespan data. Second, we did not evaluate durability after washout; health span endpoints were not included; and PICRUSt2 outputs remain predictions without metabolomic validation (e.g., SCFA quantification). Finally, we did not measure daily water intake per mouse, so we cannot rule out the possibility that palatability differences influenced the results. Future work will incorporate per-mouse sequencing, targeted metabolomics (SCFAs/retinoids), intake logging, longitudinal follow-up, and liver histology/inflammation markers.

## Supporting information

10.1017/gmb.2026.10018.sm001 Kim et al. supplementary material Kim et al. supplementary material

## Data Availability

All data supporting the conclusions of the study are included in this article and Supplementary Material. Additional study-related data are available from the corresponding author upon reasonable request.

## Supplementary material

The supplementary material for this article can be found at http://doi.org/10.1017/gmb.2026.10018.
