# Supplementary material for: Bioactive ceramic-processed water modulates the gut microbiota and hepatic AMPK activation in SMP30 knockout mice
Source: Gut Microbiome (Camb). 2026 Jan 22;7:e3. doi: 10.1017/gmb.2026.10018 (PMC12892148; doi:10.1017/gmb.2026.10018)
Supplement: Kim et al. supplementary material [file S2632289726100188sup001.docx]

**Supplementary Material**

Title

**Bioactive ceramic-processed water modulates the gut microbiota and hepatic AMPK activation in *SMP30* knockout mice**

Authors

Dong-Hun Kim^1),#^, Soo-Nyun Choi^2),#^, Kyongman An^3)^, Ji-Hoon Kwak^4)^, Kyung-Seok Ko^1)^, and Kyu-Shik Jeong^5),6),*^

Affiliations

^1)^Groundwater Environment Research Center, Geo-Environment Research Division, Korea Institute of Geoscience and Mineral Resources, Daejeon City 34132, Republic of Korea

^2)^College of Veterinary Medicine, Kyungpook National University, Daegu City 41566, Republic of Korea

^3)^AI-Bio Convergence Research Institute, Department of Industrial AI Engineering, Graduate School of Management of Technology, Hoseo University, Asan City 31499, Republic of Korea

^4)^FM Animal Medical Center, Gimpo City 10080, Republic of Korea

^5)^Stellamed Co., LTD, Daegu City 41504, Republic of Korea

^6)^Department of Pet Industry, Adventure College, Daegu Haany University, Gyeongsan City 38610, Republic of Korea

^#^ These authors contributed equally to this work.

**^*^Corresponding author:**

Professor, Dr. Kyu-Shik Jeong

Department of Pet Industry, Adventure College, Daegu Haany University, 1 Haanydaero, Gyeongsan City 38610, Gyeongsangbuk-Do, Republic of Korea

E-mail: jeongks@dhu.ac.kr; anistemcell@gmail.com

Tel: +82-10-8812-1258

**Supplementary Tables**

Table S1. Chemical analysis of Granite Ceramic and bioactive ceramic processed water (BCP). (A) Mineral constituents of Granite Ceramic.

| **Constituent** | **Granite Ceramic** |
| --- | --- |
| Qz (quartz) | 49.5 |
| Kfs (alkalifeldspar) | 12.4 |
| Pl (Plagioclase) | 21 |
| Mu (muscovite) | 17.2 |
| Total (%) | 100 |

(B) Chemical compositions of Granite Ceramic.

| **Concentration (wt. %)** | **Granite Ceramic** |
| --- | --- |
| SiO2 | 75.91 |
| Al2O3 | 13.11 |
| Fe2O3 | 2.38 |
| MnO | 0.03 |
| CaO | 2.9 |
| MgO | 2.2 |
| Na2O | 0.65 |
| K2O | 1.97 |
| P2O5 | 0.07 |
| TiO2 | 0.24 |
| Cr2O3 | 0.03 |
| LOl | 0.51 |

| **Element (ppm)** | **Granite Ceramic** |
| --- | --- |
| Ba | 327 |
| Zr | 41.2 |
| Ni | 45.2 |
| Ga | 101 |
| Hf | 1 |
| Nb | 10.2 |
| Rb | 84.7 |
| U | 3.1 |
| Th | 15.4 |
| Cu | 13.8 |
| Mo | 2.6 |
| Sb | 0.5 |

(C) Chemical element comparison: MW vs. BCP.

| **Element (ppb)** | **MW** | **BCP** |
| --- | --- | --- |
| Na | 9693.1 | 10807.5 |
| Mg | 4837.7 | 2573.1 |
| K | 1663.8 | 4540.2 |
| Ca | 5569 | 3752.2 |
| P | 116.3 | 0 |
| Ba | 0.4 | 52.2 |
| Ga | 0.1 | 15 |
| Rb | 2.5 | 12.3 |
| Sb | 0.1 | 0.3 |
| Mo | 1.9 | 0 |
| U | 0.2 | 0 |
| Nb | 0.1 | 0 |
| Ti | 0 | 0 |
| Zr | 0 | 0 |
| Ni | 0 | 0 |
| Hf | 0 | 0 |
| Th | 0 | 0 |
| Cu | 0 | 0 |

※ MW-VitC: MW supplemented with 1.5 g/L of vitamin C

Table S2. The alpha diversity indices of different drinking water treated SMP30 KO mice fecal microbiota.

| Group | Stage | Sample | Observed Features | Shannon | Simpson | Pielou's Evenness |
| --- | --- | --- | --- | --- | --- | --- |
| Young (6-week-old) | 0 week | MW | 101 | 4.491 | 0.797 | 0.973 |
|  |  | MW-vitC | 108 | 4.522 | 0.743 | 0.966 |
|  |  | BCP | 111 | 4.571 | 0.784 | 0.971 |
|  | 8 week | MW | 94 | 4.393 | 0.758 | 0.967 |
|  |  | MW-vitC | 92 | 4.395 | 0.792 | 0.972 |
|  |  | BCP | 87 | 4.273 | 0.692 | 0.957 |
| Aged (20-week-old) | 0 week | MW | 72 | 4.130 | 0.772 | 0.966 |
|  |  | MW-vitC | 101 | 4.458 | 0.752 | 0.966 |
|  |  | BCP | 88 | 4.335 | 0.777 | 0.968 |
|  | 10 week | MW | 58 | 3.730 | 0.525 | 0.919 |
|  |  | MW-vitC | 75 | 4.095 | 0.671 | 0.948 |
|  |  | BCP | 75 | 4.126 | 0.708 | 0.956 |

**Supplementary Figure**


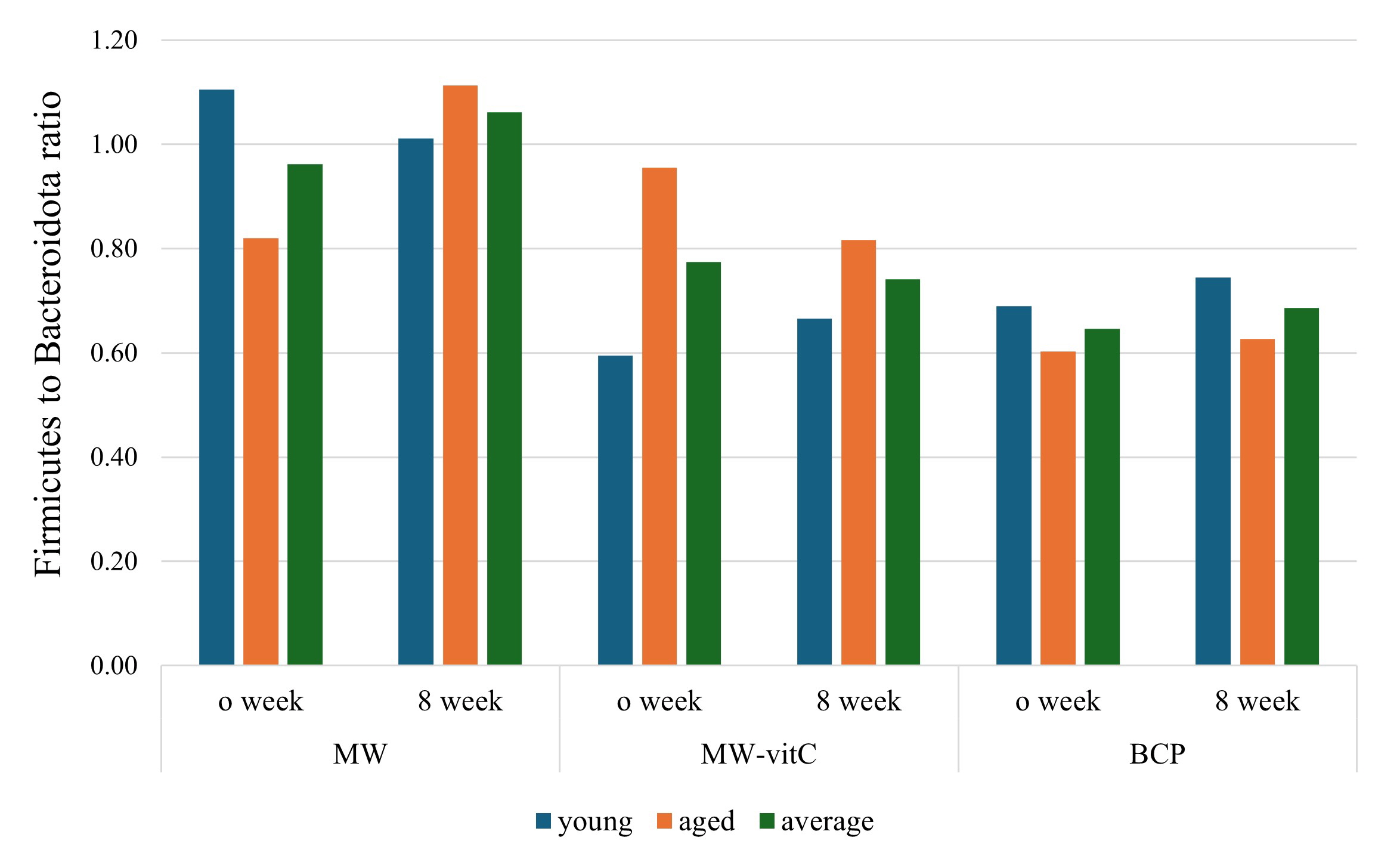


Figure S1. The Firmicutes/Bacteroidetes ratio (F/B ratio) in young and aged mice under different drinking water exposure.
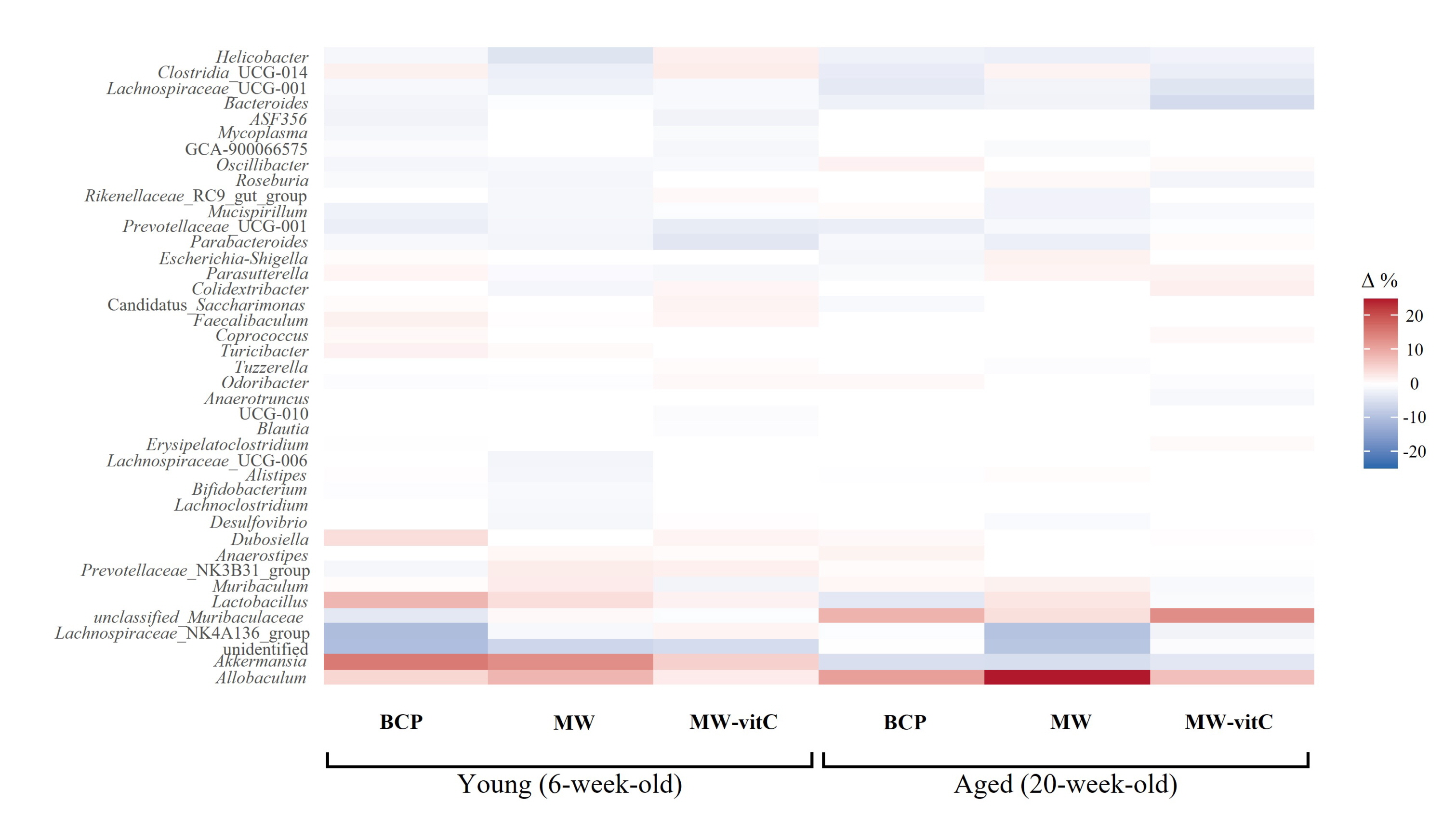


Figure S2. Heatmap of genus-level differences in relative abundance (Δ = after − before, percentage points) across young and aged groups and water regimens (BCP, MW, MW-vitC). Rows (genera) are ordered by hierarchical clustering for visualization. Positive values denote increases, negative values denote decreases.
